# Supplementary material for: MINN: A metabolic-informed neural network for integrating omics data into genome-scale metabolic modeling
Source: Comput Struct Biotechnol J. 2025 Aug 7;27:3609–17. doi: 10.1016/j.csbj.2025.08.004 (PMC12359237; doi:10.1016/j.csbj.2025.08.004)
Supplement: MMC — This supplementary material provides extended explanations and details that complement the main manuscript, including a Related Work section on genome-scale metabolic models, omics data integration, and hybrid mechanistic-data-driven approaches. It contains additional results, statistical significance tests, additional details on computational settings, and hyperparameter details. Additional figures show optimization strategies, while a step-by-step toy example guides the reader through the MINN architecture. [file mmc1.pdf]

Supplementary Materials

Gabriele Tazza, Francesco Moro, Dario Ruggeri, Bas Teusink, László Vidács

August 4, 2025

1 Related Work

Given the multidisciplinary nature of this work, we considered it important to provide a common starting point for the main topics covered, namely: Genome-Scale Metabolic Models and Flux Balance Analysis, Omics Data Integration, and Hybrid mechanistic and data-driven modeling.

1.1 Genome-Scale Metabolic Models and Flux Balance Analysis

A genome-scale metabolic model (GEM) is a comprehensive reconstruction of an organism's metabolic network, representing the full metabolic capacity encoded by its genome. It serves as a structured knowledge base, integrating information on genes, proteins, enzymes, and metabolic pathways [Monk et al., 2014, O'Brien et al., 2015]. GEMs have been primarily reconstructed for microorganisms, but models also exist for multicellular organisms, including humans. A typical microbial GEM contains hundreds or even thousands of reactions and metabolites, increasing in complexity for multicompartment systems like yeast [Somerville et al., 2022]. To analyze such large models, a commonly used method is Flux Balance Analysis (FBA), which relies on the assumption of steady state or balanced growth [Orth et al., 2010, Bruggeman et al., 2020]. Under these conditions, the concentrations of metabolites remain constant over time, and the rates of production and consumption are balanced across all reactions. GEMs can therefore be formulated only in terms of reaction rates and treated as linear programming problems. FBA uses this framework to predict the metabolic behavior of the organism by optimizing, given the stoichiometric constraints, a specific objective function: commonly biomass production or, in biotechnological contexts, the yield of a desired product. However, stoichiometric constraints alone are often insufficient to determine a realistic flux distribution. To improve predictive accuracy, FBA requires context-specific inputs—such as growth medium composition—typically incorporated by constraining exchange fluxes. These constraints may be derived from experimental measurements, assumptions about nutrient uptake kinetics, or a combination of both [Palsson, 2015].

1.2 Omics Data Integration in Genome-Scale Metabolic Models and Flux Balance Analysis

GEMs are reconstructed primarily from genomic information, encoding the metabolic network of an organism. A GEM can be tailored to represent different cell strains by including or excluding reactions based on the presence or absence of genes encoding the relevant metabolic enzymes [Thiele and Palsson, 2010]. Beyond genomics, a broad range of omics data (e.g., transcriptomics, proteomics, metabolomics, and fluxomics) can significantly enhance the accuracy and predictive capacity of GEMs [Machado and Herrgård, 2014, Yizhak et al., 2010]. The challenge lies in translating these data into metabolic fluxes. For enzyme-catalyzed reactions, the reaction rate is typically described by:

$$v = k_{cat} \cdot e \cdot f(s, p) \tag{1}$$

where:

- v is the reaction's flux;
- k_{cat} is the catalytic constant, or turnover number, which represents the number of substrate molecules converted to product per enzyme molecule per unit time when the enzyme is fully saturated with substrate, i.e., the enzyme efficiency;

- e is the enzyme concentration;
- $f(s,p)$ is a (often nonlinear) function of the concentrations of substrates s and products p, and the corresponding affinity parameters.

It is worth also noting that, while genomic, transcriptomic, and proteomic data provide rich layers of information, only features that can be explicitly linked to metabolic reactions can be directly integrated into GEMs [Liebermeister et al., 2014]. As a result, much of the broader cellular context captured by these datasets, including regulatory, structural, or signaling components, is typically excluded from the model.

Transcriptomics data, while often weakly correlated with actual protein levels or enzymatic activity [Ponomarenko et al., 2023], remain useful to identify active metabolic genes and infer condition-specific pathway activation. When comparing multiple conditions, transcriptome profiles may suggest shifts in metabolic strategy. Several frameworks have been developed to incorporate transcriptomics and gene co-expression data into GEMs [Machado and Herrgård, 2014, Paklao et al., 2023, Zampieri et al., 2023]. Transcriptomic data have also enabled the development of metabolism and gene expression models (ME-models), which explicitly couple metabolic reactions with the expression of the genes encoding the corresponding enzymes [O'Brien et al., 2013, Lloyd et al., 2018]. These models account for the transcriptional and translational cost of enzyme production and provide a mechanistic link between gene expression and flux capacity.

To address the challenge of nonlinear and context-dependent relationships between transcript levels and metabolic fluxes, some methods avoid imposing direct constraints and instead seek to maximize the consistency or correlation between gene expression and flux predictions [Zur et al., 2010, Pandey et al., 2019a,b, Alghamdi et al., 2021].

Proteomics data provide a closer proxy for metabolic capability. Presence or absence of specific enzymes can directly constrain which reactions are allowed under given conditions. Assuming enzyme saturation ($f(s,p) = 1$), enzyme levels scaled by k_{cat} values provide upper bounds for fluxes (v_{max}). These catalytic parameters can be obtained from databases such as BRENDA [Chang et al., 2021] and SABIO-RK [Wittig et al., 2018], or estimated using statistical approaches or enzyme-kinetic models [Singh et al., 2024, Diamataris et al., 2023]. Although these resources are growing rapidly, retrieving the relevant kinetic parameters remains a semi-automated process that often requires manual curation to ensure accuracy and model compatibility. Building on these concepts, more complex model formulations have been developed, such as enzyme-constrained GEMs (ecGEMs), which treat metabolism as a problem of protein budgeting under limited cellular capacity [Chen et al., 2024, Salvy and Hatzimanikatis, 2020]. Proteome-constrained models (pcGEMs) further account for the resource cost of enzyme production [Grigaitis et al., 2021, Elsemman et al., 2022]. While building these models often requires custom pipelines, tools such as GECKO [Chen et al., 2024] and sEnz [van den Bogaard et al., 2024] are making these tasks increasingly standardized and accessible.

Metabolomics data offer insights into both the structure and dynamics of metabolism. Although metabolite concentrations cannot be directly used in GEMs due to the steady-state assumption and lack of proportionality between concentrations and fluxes, their presence or absence can indicate pathway activity. Time-series measurements of extracellular metabolite levels can be converted into flux constraints for exchange reactions, allowing us to tune the GEMs to match observed uptake or secretion patterns Henriques et al. [2021]. Moreover, when quantitative metabolomics data are available for the reagents of a reaction, thermodynamic constraints can be imposed on its direction, further narrowing the feasible flux space and improving biological realism [Niebel et al., 2019, Pandey et al., 2019a,b].

Fluxomics data: isotope-labeling experiments (e.g., growth in ^{13}C-glucose medium) allow direct estimation of intracellular flux distributions via Metabolic Flux Analysis (MFA) [Antoniewicz, 2021]. MFA uses ^{13}C metabolomics data to fit simplified metabolic models, enabling the estimation of intracellular fluxes based on the observed labeling patterns. These experimentally derived fluxes can be used to constrain specific reactions or to find the flux profile that best fits the measured data.

Multi-omics data : The simultaneous integration of diverse omics layers yields a more holistic view of the cellular state. This systems-level approach is especially valuable for understanding dynamic or context-dependent responses, as it captures the interactions between different biological processes [Lu et al., 2018]. Tools like the IOMA (Integrative Omics-Metabolic Analysis) framework facilitate the incorporation of diverse omics layers into GEMs to enhance predictive fidelity [Yizhak et al., 2010].

In summary, omics data integration represents a powerful way to contextualize and refine GEMs, improving their ability to simulate real-world biological behavior. However, current methods remain limited by a lack of standardization and automation. Moreover, mechanistic integration is restricted to metabolic features explicitly

represented in GEMs, meaning that valuable context from a broader view of cellular processes is still usually excluded.

1.3 Integrating FBA and Machine Learning for Enhanced Metabolic Predictions

In the previous sections, we discussed how FBA is a powerful approach to exploit the information stored in GEMs to predict the metabolic behavior of cells. However, FBA has at least four main limitations. First, its predictive power heavily depends on the amount of experimental measurements of exchange fluxes. Second, incorporating multi-omics data is challenging, because all measurements must be converted into fluxes, a process that usually requires iterative steps of time-consuming manual curation. Third, FBA and GEMs focus solely on metabolism and typically do not link it to the general status of the cell. Finally, FBA predicts flux distributions that tend to maximize the yield on the limiting substrates [van Pelt-KleinJan et al., 2021], often missing to capture "high rate-low yield" solutions [Elsemman et al., 2022].

In recent years, with the increasing availability of high-throughput technologies and data, ML has gained popularity as a valid alternative to mechanistic-based approaches [Wytock and Motter, 2018, Gonçalves et al., 2023, Al et al., 2024]. The success of ML lies in its ability to find patterns in the data without making any mechanistic assumptions. However, a main drawback is that ML requires a high volume of data to train models successfully, and in many biology-related domains, datasets of suitable size are rare. In particular, experiments in microbial physiology tend to be one, if not two, orders of magnitude smaller than what ML requires. Moreover, ML behaves mostly as a black-box model, making it difficult to extract mechanistic understanding from its results. On the other hand, this black-box nature makes ML more amenable than mechanistic models for integrating diverse data sources, even those for which there is no clear understanding of their connections.

Therefore, it seems natural to integrate these two approaches to overcome each other's limitations and exploit their strengths. In recent years, as reviewed in Sahu et al. [2021] and Zampieri et al. [2019], there have been many attempts to integrate these methods. Sahu et al. [2021] categorize these works into two groups: ML as input of FBA Dai et al. [2018], Kim et al. [2016], Morrissey et al. [2025] and FBA as input of ML Magazzù et al. [2021], Culley et al. [2020]. This division highlights that these methods do not truly integrate ML and FBA but rather concatenate them, using them in two distinct steps. To the best of our knowledge, only three works presented hybrid models that genuinely integrate FBA and ML: Faure et al. [2023], Hasibi et al. [2024], and Alghamdi et al. [2021]. The first introduces Artificial Metabolic Neural Networks (AMNs), which are Neural Networks that use FBA constraints to refine their solution and to regularize the network. This is achieved through a Mechanistic Layer, representing the structure of the mechanistic model inside the NN, and a custom loss function, similar to those of other Knowledge Informed Neural Networks (e.g. Physics-Informed Neural Network [Cuomo et al., 2022]). When using FBA alone for growth rate prediction, nutrient uptake fluxes often need manual adjustment to match experimental growth rates. This process can involve labor-intensive experiments or unsystematic "trial-and-error" adjustments, which may introduce arbitrary assumptions to align the model with observed data. The hybrid AMN framework proposed by Faure et al. [2023] addresses these challenges by embedding mechanistic information into neural networks, providing a more systematic approach. The second presents FlowGAT, which integrates the structure of the GEM and the solution of FBA in a Graph Attention Network (GAT) to predict the gene essentiality. The third method, scFEA, combines single-cell transcriptomics with FBA-inspired constraints using a Graph Neural Network. Like AMNs, scFEA treats flux balance as a soft constraint in the loss function, but it also includes a term that explicitly maximizes the agreement between gene expression and predicted fluxes.

The MINN models presented here follow the blueprint of AMNs and, in line with the approach presented in Faure et al. [2023], represent a true hybrid model, integrating FBA constraints and multi-omics data to improve predictions of fluxes. One key feature of MINN is that it incorporates omics data not only for elements (genes, proteins, etc.) directly linked to metabolic activity, but also those representative of the broader cellular context, and it leaves it to the neural network component to learn the complex relationships between all omics layers and metabolic fluxes. However, as a possible future development, GPRs could be embedded directly into the network architecture or in the loss function of a MINN, strengthening the mechanistic link between omics data and flux predictions and further leveraging the structure encoded in the GEM.

A first implementation of the model was presented in Tazza et al. [2024]. Here we further develop this architecture and test different solutions to mitigate conflicts that emerged between the data-driven and the mechanistic objectives.

2 Additional Figures: Hybrid Optimization Strategies for Data-Driven and Mechanistic Integration

Figure 1 shows the bound on mechanistic loss which penalizes solutions that stray too far from the mechanistic loss threshold, encouraging the model to respect mechanistic constraints during training.

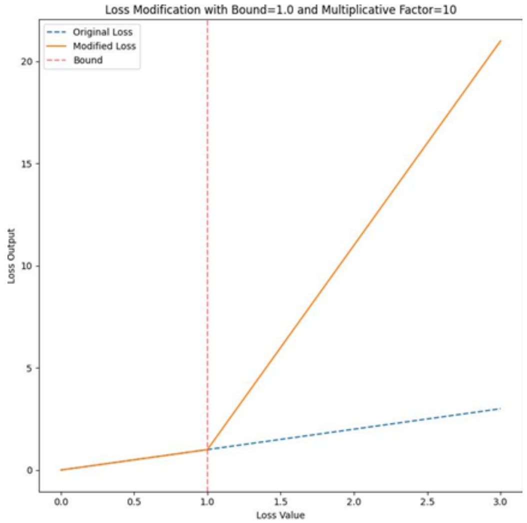

Figure 1: Illustration of the mechanistic loss bound application. The original loss (blue dashed line) remains linear, while the modified loss (orange line) increases steeply after surpassing the bound (red vertical line). This demonstrates how the bound prevents the mechanistic loss from exceeding a set threshold by applying a multiplicative factor beyond this limit.

Figure 2 illustrates how the scheduler dynamically adjusts the weight of the losses over the course of training, allowing the model to optimize both objectives.

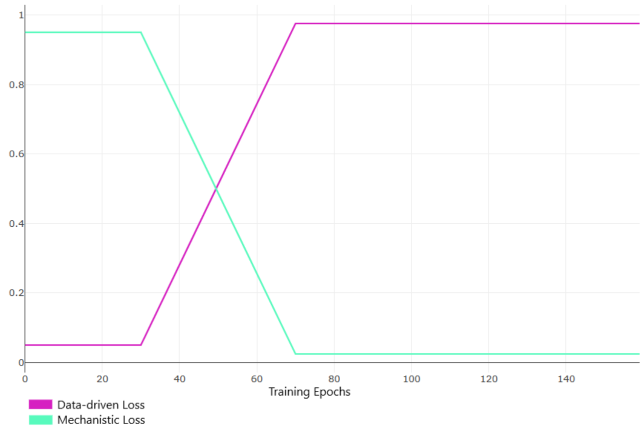

Figure 2: Visualization of the dynamic loss scheduler. The scheduler adjusts the weight of the mechanistic and data-driven losses throughout training, starting with the mechanistic objective and gradually transitioning to prioritize the data-driven objective. This ensures the model initially aligns with mechanistic constraints before focusing on data-driven optimization.

3 Additional Results

3.1 Other GEMs comparison

Here we present the results of the analysis to explore the role of the GEM in the models' performance. As shown in Table 1, we divided the results in two parts. The first one contains different GEMs in terms of dimension. Here the GEM is always iAF1260, but reduced in different ways described in details in the Section "GEM preparation". We also built a MINN with the *E. coli* core model (e_coli_core), to test the smallest version available on BiGG `http://bigg.ucsd.edu/`. The GEM's dimension can affect the complexity of the NN block in the MINN. Having a layer with many neurons can cause both overfitting and a higher computational time. At the same time, excessively reducing the GEM can decrease the flexibility in the optimization of FBA constraints and make the GEM less representative of the experimental context considered. The first section of Table 1 shows how the FVA reduction has better performances, in terms of metrics and L_2, and lower computational time (24h vs 44h) than the full GEM. FBA reduction, instead, performs slightly worse than FVA, but it halves the computational time. On the other hand, the e_coli_core GEM performs drastically worse than all the others, making this GEM unfit for this task.

The results show a trade-off between GEM size and computational efficiency. A stricter reduction, such as FBA reduction, shortens the computational time, but at the cost of slightly worse metrics and less reliable flux distribution. While this compromise may not be ideal for small models, it can be helpful for large GEMs, such as yeast or microbial communities, where computational feasibility is critical. In such cases, sacrificing some predictive power in exchange for reasonable runtimes can be a proper trade-off.
Additionally, the poor performance of the e_coli_core model highlights the need for an adequate dimension of the GEM, reinforcing the idea that excessively small models may not represent the experimental context of interest.

	ISHII				
GEM	R^2	MAE	RMSE	NE	L_2
iAF1260	0.818 ± 0.670	0.602 ± 0.653	1.084 ± 0.813	0.417 ± 0.268	$4.05 \cdot 10^{-5} \pm 1.57 \cdot 10^{-4}$
iAF1260 *FVA-reduced*	0.950 ± 0.055	0.473 ± 0.480	0.678 ± 0.653	0.272 ± 0.280	$8.75 \cdot 10^{-5} \pm 2.95 \cdot 10^{-4}$
iAF1260 *FBA-reduced*	0.950 ± 0.048	0.509 ± 0.518	0.730 ± 0.719	0.289 ± 0.295	$1.26 \cdot 10^{-4} \pm 2.84 \cdot 10^{-4}$
e_coli_core	0.061 ± 0.099	4.647 ± 9.959	19.46 ± 65.30	7.584 ± 26.70	$6.04 \cdot 10^{5} \pm 2.55 \cdot 10^{6}$
iAF1260 *FVA-reduced*	0.956 ± 0.056	0.512 ± 0.596	0.759 ± 0.815	0.285 ± 0.337	$4.27 \cdot 10^{-5} \pm 1.3 \cdot 10^{-4}$
iNF517 *FVA-reduced*	0.954 ± 0.057	0.546 ± 0.612	0.801 ± 0.855	0.304 ± 0.358	$5.24 \cdot 10^{-5} \pm 1.02 \cdot 10^{-4}$

Table 1: Performance comparison between different GEMs. Metrics average and standard deviation over 29 leave-one-out splits.

The second part includes a comparison between two GEMs representing two different bacteria, namely *E.coli* and *Lactococcus lactis subsp. cremoris*. The aim of this analysis is to explore how relevant the nature of the GEM is in the MINN architecture. We want to investigate if the regularization that improves the predictive power of the MINN w.r.t. a classical ML approaches is based on a relevant biological information injected in the model through the mechanistic layer, or it's simply a random type of regularization such as Dropout [Srivastava et al., 2014]. Since the *L.cremoris* GEM (iNF517) does not have some of the reactions present in the ISHII dataset, in order to have a fair comparison with the *E.coli* GEM (iAF1260), we reduced the number of fluxes to only those in common between *L.cremoris* and the ISHII dataset. As expected, using a GEM which belongs to another bacterium worsen the prediction performance and also the quality of the predicted flux distribution.

However, the difference in performance between the iAF1260 *FVA-reduced* and iNF517 *FVA-reduced* GEMs is not particularly large. One possible explanation is that the measured fluxes in ISHII dataset belong to the central carbon metabolism, which is highly conserved between both bacteria, reducing the impact of GEM differences. Another factor could be the neural network data-driven component, which may help compensate for discrepancies between GEMs, reducing their effect on predictive performance.

While further investigation is needed, these results suggest that the nature of the GEM plays an important role in the MINN framework. The mechanistic layer likely contributes with biologically relevant information beyond acting as a generic regularization mechanism.

3.2 Tests of Significance

To assess whether the performance differences observed in our experiments are statistically significant or potentially due to chance, we conducted a series of Wilcoxon signed-rank tests. For each of the main models comparison, we computed four separate p-values, one for each evaluation metrics used. To combine these into a single measure of significance, we employed Fisher's method, as implemented in the scipy library. We first compared MINN-c-balanced to pFBA (Table 2 of the main manuscript). The final combined p-value resulting from Fisher's method is $6.09 \cdot 10^{-18}$, allowing us to confidently state that the performance difference is statistically significant. We then compared MINN-c-balanced to the pure neural network (also Table 2), obtaining a combined p-value of 0.0009, which also confirms statistical significance. In contrast, the comparison between MINN-c-balanced and the Random Forest model showed a final combined p-value of 0.97, meaning we cannot reject the null hypothesis and conclude that there is a significant difference in performance between the two models. Lastly, for the comparison between the reservoir model and pFBA reported in Table 4 of the main manuscript, the final combined p-value is $2.34 \cdot 10^{-19}$, strongly indicating a statistically significant improvement in performance. These results highlight the potential of hybrid approaches such as MINN-based methods, but also indicate that their advantage over traditional ML models like Random Forests may vary depending on the context or the dataset and remains an open question for future work.

4 Additional details on Computational Setup

4.1 Computational Settings

In this section, we report the runtime of the main experiments conducted in our analysis. The goal is to provide practical insights into the computational cost of each method, guiding practical adoption. All experiments were managed using the ClearML framework. While ClearML introduces a slight overhead due to logging and management features, this does not significantly affect the runtimes provided. For methods presented in Gonçalves et al., quantitative runtime data are not available in the original publication. Therefore, we provide qualitative intervals. For MINN-based approaches and other models we developed, we report quantitative runtime measurements obtained directly from our experimental pipeline. All experiments were executed on a machine equipped with an NVIDIA GeForce RTX 2080 Ti GPU (11GB memory), 20 CPU cores, and 126 GB of RAM. Full software configurations, including the Docker image and package dependencies, are available in the associated code repository to ensure full reproducibility. A summary of all runtime estimates is provided in Table 2.

Method	GEM	Evaluation pipeline	Runtime
pFBA	iAF1260	Test only	<1min
NN	NA	Train + Val + Test	<5h
RF	NA	Train + Val + Test	<2h
MINN-c-balanced	iAF1260	Train + Val + Test	44h
MINN-c-balanced	iAF1260 FVA-reduced	Train + Val + Test	24h
MINN-c-balanced	iAF1260 FBA-reduced	Train + Val + Test	12h
MINN-c-balanced	e_coli_core	Train + Val + Test	9.45h
MINN-c-balanced	iNF517 FVA-reduced	Train + Val + Test	14.30h
reservoir + pFBA	iAF1260 FBA-reduced	(Pretrain) + Train + Val + Test	(6.40h) + 6.16h

Table 2: Runtime details for baselines and MINN-based methods

4.2 Hyperparameters details

Table 3 shows a detailed list of the hyperparameter search spaces used during the tuning process for each of the methods presented in this work. As described in the main manuscript, hyperparameter optimization is performed in the inner loop of the evaluation pipeline, which follows a 5-fold cross-validation scheme. We used random search to explore the search space. Some hyperparameters were optimized, while others were fixed based on prior knowledge from Gonçalves et al. [2023]. Specifically, we fixed the number of epochs at 100, the batch size at 5, and we always used the Adam optimizer. As shown in Table 3, we defined different search spaces for the value of the constant c, the parameter balancing the data-driven and mechanistic losses (for the last phase of the

schedulers models), the hidden layer size of the neural network, the dropout rate, the learning rate, and the $L2$ regularization term.

5 MINN architecture: toy example

Here we present a toy example (Figure 3) that shows step by step how the MINN architecture works. Starting from a single input sample, we walk through the key components of the model: from omics feature concatenation, to the neural network prediction, and finally to the mechanistic refinement using FBA constraints.

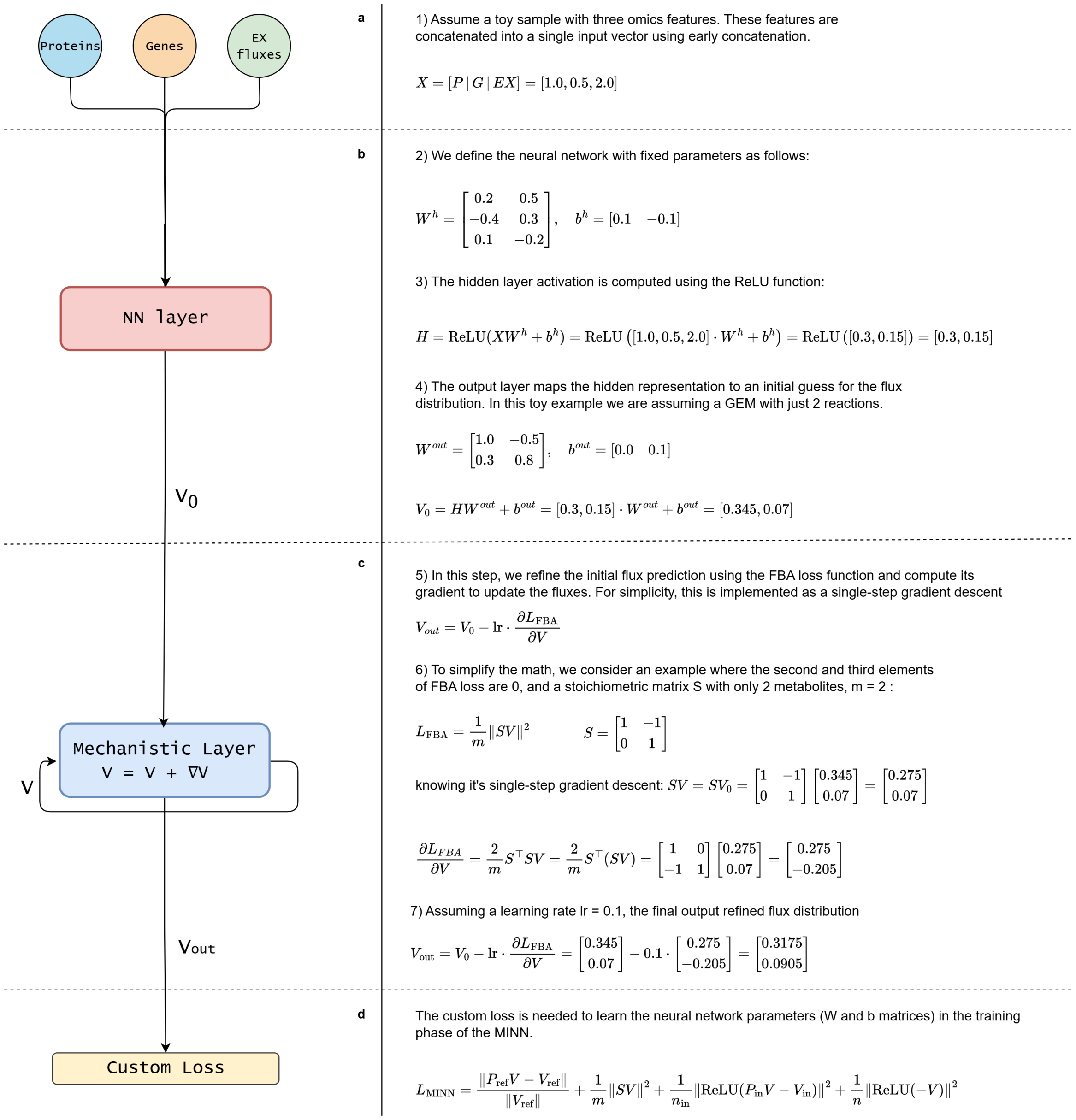

Figure 3: Toy example illustrating the workflow of the MINN architecture. **(a)** Omics features (proteomics, transcriptomics, exchange fluxes) are concatenated into a single input vector X. **(b)** A feedforward neural network maps X to an initial flux prediction V_0 using learned weights. **(c)** A mechanistic layer refines V_0 via one step of gradient descent, enforcing FBA constraints, and outputs the predicted flux distribution V_{out}. **(d)** A custom loss combining prediction error and FBA constraints is used to update the neural network during training.

	Hyperparameter Search Space					
Method	c	Final Loss Balance Weight	Hidden Size	Learning Rate	Dropout Rate	L2
MINN-MSE-base	NA	NA	{200, 250, 300}	{0.0002, 0.0005, 0.0007}	{0.1, 0.25, 0.5}	{0, 0.0001, 0.001}
MINN-unbalanced	NA	NA	{200, 250, 300}	{0.0002, 0.0005, 0.0007}	{0.1, 0.25, 0.5}	{0, 0.0001, 0.001}
MINN-c-balanced	{10, 20, 30, 40, 50}	NA	{200, 250, 300}	{0.0002, 0.0005, 0.0007}	{0.1, 0.25, 0.5}	{0, 0.0001, 0.001}
MINN-bound	{10, 20, 30, 40, 50}	NA	{200, 250, 300}	{0.0002, 0.0005, 0.0007}	{0.1, 0.25, 0.5}	{0, 0.0001, 0.001}
MINN-scheduler	NA	[0.8, 1]	{200, 250, 300}	{0.0002, 0.0005, 0.0007}	{0.1, 0.25, 0.5}	{0, 0.0001, 0.001}
MINN-scheduler-bound	NA	[0.8, 1]	{200, 250, 300}	{0.0002, 0.0005, 0.0007}	{0.1, 0.25, 0.5}	{0, 0.0001, 0.001}
MINN-reservoir + pFBA	NA	NA	{200, 250, 300}	{0.0002, 0.0005, 0.0007}	{0.1, 0.25, 0.5}	{0, 0.0001, 0.001}

Table 3: Hyperparameter search spaces used during tuning for each method. Square brackets denote discrete sets of values, while curly brackets indicate continuous intervals.

References

Jonathan Monk, Juan Nogales, and Bernhard O. Palsson. Optimizing genome-scale network reconstructions. *Nature Biotechnology*, 32(5):447–452, May 2014. ISSN 1546-1696. doi: 10.1038/nbt.2870. URL `https://www.nature.com/articles/nbt.2870`. Publisher: Nature Publishing Group.

Edward J. O'Brien, Jonathan M. Monk, and Bernhard O. Palsson. Using Genome-scale Models to Predict Biological Capabilities. *Cell*, 161(5):971–987, May 2015. ISSN 00928674. doi: 10.1016/j.cell.2015.05.019. URL `https://linkinghub.elsevier.com/retrieve/pii/S0092867415005681`.

Vincent Somerville, Pranas Grigaitis, Julius Battjes, Francesco Moro, and Bas Teusink. Use and limitations of genome-scale metabolic models in food microbiology. *Current Opinion in Food Science*, 43:225–231, February 2022. ISSN 2214-7993. doi: 10.1016/j.cofs.2021.12.010. URL `https://www.sciencedirect.com/science/article/pii/S2214799321001703`.

Jeffrey D. Orth, Ines Thiele, and Bernhard Ø Palsson. What is flux balance analysis? *Nature Biotechnology*, 28 (3):245–248, March 2010. ISSN 1546-1696. doi: 10.1038/nbt.1614. URL `http://www.nature.com/articles/nbt.1614`. Number: 3 Publisher: Nature Publishing Group.

Frank J Bruggeman, Robert Planqué, Douwe Molenaar, and Bas Teusink. Searching for principles of microbial physiology. *FEMS Microbiology Reviews*, 44(6):821–844, November 2020. ISSN 0168-6445. doi: 10.1093/femsre/fuaa034. URL `https://doi.org/10.1093/femsre/fuaa034`.

B. O. Palsson. Systems biology: Constraint-based reconstruction and analysis, 2015.

Ines Thiele and Bernhard Ø Palsson. A protocol for generating a high-quality genome-scale metabolic reconstruction. *Nature Protocols*, 5(1):93–121, January 2010. ISSN 1750-2799. doi: 10.1038/nprot.2009.203. URL `https://www.nature.com/articles/nprot.2009.203`. Publisher: Nature Publishing Group.

Daniel Machado and Markus Herrgård. Systematic Evaluation of Methods for Integration of Transcriptomic Data into Constraint-Based Models of Metabolism. *PLOS Computational Biology*, 10(4):e1003580, April 2014. doi: 10.1371/journal.pcbi.1003580.

Keren Yizhak, Tomer Benyamini, Wolfram Liebermeister, Eytan Ruppin, and Tomer Shlomi. Integrating quantitative proteomics and metabolomics with a genome-scale metabolic network model. *Bioinformatics*, 26 (12):i255–i260, June 2010. ISSN 1367-4803. doi: 10.1093/bioinformatics/btq183. URL `https://doi.org/10.1093/bioinformatics/btq183`.

Wolfram Liebermeister, Elad Noor, Avi Flamholz, Dan Davidi, Jörg Bernhardt, and Ron Milo. Visual account of protein investment in cellular functions. *Proceedings of the National Academy of Sciences*, 111(23):8488–8493, June 2014. doi: 10.1073/pnas.1314810111. URL `https://www.pnas.org/doi/abs/10.1073/pnas.1314810111`. Publisher: Proceedings of the National Academy of Sciences.

Elena A. Ponomarenko, George S. Krasnov, Olga I. Kiseleva, Polina A. Kryukova, Viktoriia A. Arzumanian, Georgii V. Dolgalev, Ekaterina V. Ilgisonis, Andrey V. Lisitsa, and Ekaterina V. Poverennaya. Workability of mRNA Sequencing for Predicting Protein Abundance. *Genes*, 14(11):2065, November 2023. ISSN 2073-4425. doi: 10.3390/genes14112065. URL `https://www.mdpi.com/2073-4425/14/11/2065`. Number: 11 Publisher: Multidisciplinary Digital Publishing Institute.

Thummarat Paklao, Apichat Suratanee, and Kitiporn Plaimas. ICON-GEMs: integration of co-expression network in genome-scale metabolic models, shedding light through systems biology. *BMC Bioinformatics*, 24(1):492, December 2023. ISSN 1471-2105. doi: 10.1186/s12859-023-05599-0. URL `https://doi.org/10.1186/s12859-023-05599-0`.

Guido Zampieri, Stefano Campanaro, Claudio Angione, and Laura Treu. Metatranscriptomics-guided genome-scale metabolic modeling of microbial communities. *Cell Reports Methods*, 3(1), January 2023. ISSN 2667-2375. doi: 10.1016/j.crmeth.2022.100383. URL `https://www.cell.com/cell-reports-methods/abstract/S2667-2375(22)00288-0`. Publisher: Elsevier.

Edward J O'Brien, Joshua A Lerman, Roger L Chang, Daniel R Hyduke, and Bernhard Ø Palsson. Genome-scale models of metabolism and gene expression extend and refine growth phenotype prediction. *Molecular Systems Biology*, 9(1):693, January 2013. ISSN 1744-4292. doi: 10.1038/msb.2013.52. URL https://www.embopress.org/doi/full/10.1038/msb.2013.52. Publisher: John Wiley & Sons, Ltd.

Colton J. Lloyd, Ali Ebrahim, Laurence Yang, Zachary A. King, Edward Catoiu, Edward J. O'Brien, Joanne K. Liu, and Bernhard O. Palsson. COBRAme: A computational framework for genome-scale models of metabolism and gene expression. *PLOS Computational Biology*, 14(7):e1006302, July 2018. ISSN 1553-7358. doi: 10.1371/journal.pcbi.1006302. URL https://journals.plos.org/ploscompbiol/article?id=10.1371/journal.pcbi.1006302. Publisher: Public Library of Science.

Hadas Zur, Eytan Ruppin, and Tomer Shlomi. iMAT: an integrative metabolic analysis tool. *Bioinformatics*, 26(24):3140–3142, December 2010. ISSN 1367-4803. doi: 10.1093/bioinformatics/btq602. URL https://doi.org/10.1093/bioinformatics/btq602.

Vikash Pandey, Daniel Hernandez Gardiol, Anush Chiappino-Pepe, and Vassily Hatzimanikatis. TEX-FBA: A constraint-based method for integrating gene expression, thermodynamics, and metabolomics data into genome-scale metabolic models, January 2019a. URL https://www.biorxiv.org/content/10.1101/536235v1. Pages: 536235 Section: New Results.

Vikash Pandey, Noushin Hadadi, and Vassily Hatzimanikatis. Enhanced flux prediction by integrating relative expression and relative metabolite abundance into thermodynamically consistent metabolic models. *PLOS Computational Biology*, 15(5):e1007036, May 2019b. ISSN 1553-7358. doi: 10.1371/journal.pcbi.1007036. URL https://journals.plos.org/ploscompbiol/article?id=10.1371/journal.pcbi.1007036. Publisher: Public Library of Science.

Norah Alghamdi, Wennan Chang, Pengtao Dang, Xiaoyu Lu, Changlin Wan, Silpa Gampala, Zhi Huang, Jiashi Wang, Qin Ma, Yong Zang, Melissa Fishel, Sha Cao, and Chi Zhang. A graph neural network model to estimate cell-wise metabolic flux using single-cell RNA-seq data. *Genome Research*, 31(10):1867–1884, October 2021. ISSN 1088-9051, 1549-5469. doi: 10.1101/gr.271205.120. URL http://genome.cshlp.org/lookup/doi/10.1101/gr.271205.120.

Antje Chang, Lisa Jeske, Sandra Ulbrich, Julia Hofmann, Julia Koblitz, Ida Schomburg, Meina Neumann-Schaal, Dieter Jahn, and Dietmar Schomburg. BRENDA, the ELIXIR core data resource in 2021: new developments and updates. *Nucleic Acids Research*, 49(D1):D498–D508, January 2021. ISSN 0305-1048. doi: 10.1093/nar/gkaa1025. URL https://doi.org/10.1093/nar/gkaa1025.

Ulrike Wittig, Maja Rey, Andreas Weidemann, Renate Kania, and Wolfgang Müller. SABIO-RK: an updated resource for manually curated biochemical reaction kinetics. *Nucleic Acids Research*, 46(D1):D656–D660, January 2018. ISSN 0305-1048. doi: 10.1093/nar/gkx1065. URL https://doi.org/10.1093/nar/gkx1065.

Divya Singh, Tal Robin, Michael Urbakh, and Shlomi Reuveni. High-order Michaelis-Menten equations allow inference of hidden kinetic parameters in enzyme catalysis, June 2024. URL https://www.biorxiv.org/content/10.1101/2024.06.12.598609v1. Pages: 2024.06.12.598609 Section: New Results.

Ioannis G. Diamataris, Loukas D. Peristeras, Konstantinos D. Papavasileiou, Vasilios S. Melissas, and Georgios C. Boulougouris. Statistical Inference of Rate Constants in Chemical and Biochemical Reaction Networks Using an "Inverse" Event-Driven Kinetic Monte Carlo Method. *The Journal of Physical Chemistry B*, 127(42): 9132–9143, October 2023. ISSN 1520-6106. doi: 10.1021/acs.jpcb.3c03649. URL https://doi.org/10.1021/acs.jpcb.3c03649. Publisher: American Chemical Society.

Yu Chen, Johan Gustafsson, Albert Tafur Rangel, Mihail Anton, Iván Domenzain, Cheewin Kittikunapong, Feiran Li, Le Yuan, Jens Nielsen, and Eduard J. Kerkhoven. Reconstruction, simulation and analysis of enzyme-constrained metabolic models using GECKO Toolbox 3.0. *Nature Protocols*, 19(3):629–667, March 2024. ISSN 1750-2799. doi: 10.1038/s41596-023-00931-7. URL https://www.nature.com/articles/s41596-023-00931-7. Publisher: Nature Publishing Group.

Pierre Salvy and Vassily Hatzimanikatis. The ETFL formulation allows multi-omics integration in thermodynamics-compliant metabolism and expression models. *Nature Communications*, 11(1):30, January 2020. ISSN 2041-1723. doi: 10.1038/s41467-019-13818-7. URL https://www.nature.com/articles/s41467-019-13818-7. Publisher: Nature Publishing Group.

Pranas Grigaitis, Brett G. Olivier, Tomas Fiedler, Bas Teusink, Ursula Kummer, and Nadine Veith. Protein cost allocation explains metabolic strategies in Escherichia coli. *Journal of Biotechnology*, 327:54–63, February 2021. ISSN 01681656. doi: 10.1016/j.jbiotec.2020.11.003. URL https://linkinghub.elsevier.com/retrieve/pii/S0168165620303047.

Ibrahim E. Elsemman, Angelica Rodriguez Prado, Pranas Grigaitis, Manuel Garcia Albornoz, Victoria Harman, Stephen W. Holman, Johan Van Heerden, Frank J. Bruggeman, Mark M. M. Bisschops, Nikolaus Sonnenschein, Simon Hubbard, Rob Beynon, Pascale Daran-Lapujade, Jens Nielsen, and Bas Teusink. Whole-cell modeling in yeast predicts compartment-specific proteome constraints that drive metabolic strategies. *Nature Communications*, 13(1):801, February 2022. ISSN 2041-1723. doi: 10.1038/s41467-022-28467-6. URL https://www.nature.com/articles/s41467-022-28467-6.

Samira van den Bogaard, Pedro A Saa, and Tobias B Alter. Sensitivities in protein allocation models reveal distribution of metabolic capacity and flux control. *Bioinformatics*, 40(12):btae691, December 2024. ISSN 1367-4811. doi: 10.1093/bioinformatics/btae691. URL https://doi.org/10.1093/bioinformatics/btae691.

David Henriques, Romain Minebois, Sebastián N. Mendoza, Laura G. Macías, Roberto Pérez-Torrado, Eladio Barrio, Bas Teusink, Amparo Querol, and Eva Balsa-Canto. A Multiphase Multiobjective Dynamic Genome-Scale Model Shows Different Redox Balancing among Yeast Species of the Saccharomyces Genus in Fermentation. *mSystems*, 6(4):e00260–21, August 2021. doi: 10.1128/mSystems.00260-21. URL https://journals.asm.org/doi/10.1128/mSystems.00260-21. Publisher: American Society for Microbiology.

Bastian Niebel, Simeon Leupold, and Matthias Heinemann. An upper limit on Gibbs energy dissipation governs cellular metabolism. *Nature Metabolism*, 1(1):125–132, January 2019. ISSN 2522-5812. doi: 10.1038/s42255-018-0006-7. URL https://www.nature.com/articles/s42255-018-0006-7.

Maciek R. Antoniewicz. A guide to metabolic flux analysis in metabolic engineering: Methods, tools and applications. *Metabolic Engineering*, 63:2–12, January 2021. ISSN 1096-7176. doi: 10.1016/j.ymben.2020.11.002. URL https://www.sciencedirect.com/science/article/pii/S1096717620301683.

Hongzhong Lu, Weiqiang Cao, Xiaoyun Liu, Yufei Sui, Liming Ouyang, Jianye Xia, Mingzhi Huang, Yingping Zhuang, Siliang Zhang, Henk Noorman, and Ju Chu. Multi-omics integrative analysis with genome-scale metabolic model simulation reveals global cellular adaptation of Aspergillus niger under industrial enzyme production condition. *Scientific Reports*, 8(1):14404, September 2018. ISSN 2045-2322. doi: 10.1038/s41598-018-32341-1. URL https://www.nature.com/articles/s41598-018-32341-1. Publisher: Nature Publishing Group.

Eunice van Pelt-KleinJan, Daan H. de Groot, and Bas Teusink. Understanding FBA Solutions under Multiple Nutrient Limitations. *Metabolites*, 11(5):257, May 2021. ISSN 2218-1989. doi: 10.3390/metabo11050257. URL https://www.mdpi.com/2218-1989/11/5/257. Number: 5 Publisher: Multidisciplinary Digital Publishing Institute.

Thomas P. Wytock and Adilson E. Motter. Predicting growth rate from gene expression. *Proceedings of the National Academy of Sciences*, 116(2):367–372, December 2018. ISSN 1091-6490. doi: 10.1073/pnas.1808080116. URL http://dx.doi.org/10.1073/pnas.1808080116.

Daniel M. Gonçalves, Rui Henriques, and Rafael S. Costa. Predicting metabolic fluxes from omics data via machine learning: Moving from knowledge-driven towards data-driven approaches. *Computational and Structural Biotechnology Journal*, pages 4960–4973, January 2023. doi: 10.1016/j.csbj.2023.10.002.

Serhat Al, Fatma Uysal Ciloglu, Aytac Akcay, and Ahmet Koluman. Machine learning models for prediction of escherichia coli o157:h7 growth in raw ground beef at different storage temperatures. *Meat Science*, 210:109421, April 2024. ISSN 0309-1740. doi: 10.1016/j.meatsci.2023.109421. URL http://dx.doi.org/10.1016/j.meatsci.2023.109421.

Ankur Sahu, Mary-Ann Blätke, Jędrzej Jakub Szymański, and Nadine Töpfer. Advances in flux balance analysis by integrating machine learning and mechanism-based models. *Computational and Structural Biotechnology Journal*, 19:4626–4640, 2021. ISSN 2001-0370. doi: 10.1016/j.csbj.2021.08.004. URL http://dx.doi.org/10.1016/j.csbj.2021.08.004.

Guido Zampieri, Supreeta Vijayakumar, Elisabeth Yaneske, and Claudio Angione. Machine and deep learning meet genome-scale metabolic modeling. *PLoS Computational Biology*, 15(7), July 2019. doi: 10.1371/journal.pcbi.1007084.

David Dai, Nicholas Horvath, and Jeffrey Varner. Dynamic sequence specific constraint-based modeling of cell-free protein synthesis. *Processes*, 6(8):132, August 2018. ISSN 2227-9717. doi: 10.3390/pr6080132. URL http://dx.doi.org/10.3390/pr6080132.

Minseung Kim, Navneet Rai, Violeta Zorraquino, and Ilias Tagkopoulos. Multi-omics integration accurately predicts cellular state in unexplored conditions for escherichia coli. *Nature Communications*, 7(1), October 2016. ISSN 2041-1723. doi: 10.1038/ncomms13090. URL http://dx.doi.org/10.1038/ncomms13090.

James Morrissey, Gianmarco Barberi, Benjamin Strain, Pierantonio Facco, and Cleo Kontoravdi. Next-fba: A hybrid stoichiometric/data-driven approach to improve intracellular flux predictions. *Metabolic Engineering*, March 2025. ISSN 1096-7176. doi: 10.1016/j.ymben.2025.03.010. URL http://dx.doi.org/10.1016/j.ymben.2025.03.010.

Giuseppe Magazzù, Guido Zampieri, and Claudio Angione. Multimodal regularized linear models with flux balance analysis for mechanistic integration of omics data. *Bioinformatics*, 37(20):3546–3552, May 2021. ISSN 1367-4811. doi: 10.1093/bioinformatics/btab324. URL http://dx.doi.org/10.1093/bioinformatics/btab324.

Christopher Culley, Supreeta Vijayakumar, Guido Zampieri, and Claudio Angione. A mechanism-aware and multiomic machine-learning pipeline characterizes yeast cell growth. *Proceedings of the National Academy of Sciences*, 117(31):18869–18879, July 2020. ISSN 1091-6490. doi: 10.1073/pnas.2002959117. URL http://dx.doi.org/10.1073/pnas.2002959117.

Léon Faure, Bastien Mollet, Wolfram Liebermeister, and Jean-Loup Faulon. A neural-mechanistic hybrid approach improving the predictive power of genome-scale metabolic models. *Nature Communications*, August 2023. doi: 10.1038/s41467-023-40380-0.

Ramin Hasibi, Tom Michoel, and Diego A. Oyarzún. Integration of graph neural networks and genome-scale metabolic models for predicting gene essentiality. *npj Systems Biology and Applications*, 10(1), March 2024. ISSN 2056-7189. doi: 10.1038/s41540-024-00348-2. URL http://dx.doi.org/10.1038/s41540-024-00348-2.

Salvatore Cuomo, Vincenzo Schiano Di Cola, Fabio Giampaolo, Gianluigi Rozza, Maziar Raissi, and Francesco Piccialli. Scientific machine learning through physics–informed neural networks: Where we are and what's next. *Journal of Scientific Computing*, 92(3), July 2022. ISSN 1573-7691. doi: 10.1007/s10915-022-01939-z. URL http://dx.doi.org/10.1007/s10915-022-01939-z.

Gabriele Tazza, Francesco Moro, Bas Teusink, and László Vidács. Metabolic-informed neural network for multi-omics data integration. In Jan F.M. Van Impe and Monika E. Polańska, editors, *13th International Conference on Simulation and Modelling in the Food and Bio-Industry (FOODSIM 2024)*, pages 193–197. Eurosis-ETI, 2024. ISBN 9789492859297. Publisher Copyright: © 2024, EUROSIS-ETI. All rights reserved.; 13th International Conference on Simulation and Modelling in the Food and Bio-Industry, FOODSIM 2024 ; Conference date: 07-04-2024 Through 11-04-2024.

Nitish Srivastava, Geoffrey Hinton, Alex Krizhevsky, Ilya Sutskever, and Ruslan Salakhutdinov. Dropout: A simple way to prevent neural networks from overfitting. *Journal of Machine Learning Research*, 15(56): 1929–1958, 2014. URL http://jmlr.org/papers/v15/srivastava14a.html.
